# Intranasal epinephrine effects on epinephrine pharmacokinetics and heart rate in a nasal congestion canine model

**DOI:** 10.1186/s12931-020-01343-x

**Published:** 2020-04-03

**Authors:** Richard Tuttle, Luca Popescu, Scott Hill, Amber Slanczka, Jeffrey Jankowski, Katherine Barre, Erika Krueger, Desmond Slade, Claire Croutch, Matthew Robben, Zack Mesa, Michael Mesa, Kenneth L. Dretchen

**Affiliations:** 1 MRIGlobal, 425 Volker Boulevard, Kansas City, MO 64110-2241 USA; 2 Charles River Laboratories, Inc, Wilmington, MA USA; 3 Robben Consulting, LLC, Rochester, USA; 4 Mesa Science Associates, Inc, Frederick, MD USA

**Keywords:** Epinephrine, Nasal congestion, Histamine, Vasodilation, Intranasal, Severe allergy, Anaphylaxis, Allergy

## Abstract

**Background:**

Histamine release and vasodilation during an allergic reaction can alter the pharmacokinetics of drugs administered via the intranasal (IN) route. The current study evaluated the effects of histamine-induced nasal congestion on epinephrine pharmacokinetics and heart rate changes after IN epinephrine.

**Methods:**

Dogs received 5% histamine or saline IN followed by 4 mg epinephrine IN. Nasal restriction pressure, epinephrine concentration, and heart rate were assessed. Maximum concentration (C_max_), area under plasma concentration-time curve from 1 to 90 min (AUC_1–90_), and time to reach C_max_ (T_max_) were measured. Clinical observations were documented.

**Results:**

In the 12 dogs in this study, nasal congestion occurred at 5–10 min after IN histamine administration versus no nasal congestion after IN saline. After administration of IN epinephrine, IN histamine-mediated nasal congestion was significantly reduced to baseline levels at 60, 80, and 100 min. There were no significant differences in C_max_ and AUC_1–90_ between histamine and saline groups after IN epinephrine delivery (3.5 vs 1.7 ng/mL, *p* = 0.06, and 117 vs 59 ng/mL*minutes, *p* = 0.09, respectively). After receiving IN epinephrine, the histamine group had a significantly lower T_max_ versus the saline group (6 vs 70 min, respectively; *p* = 0.02). Following IN epinephrine administration, the histamine group showed rapidly increased heart rate at 5 min, while there was a delayed increase in heart rate (occurring 30–60 min after administration) in the saline group. Clinical observations included salivation and emesis.

**Conclusion:**

IN histamine led to more rapid epinephrine absorption and immediately increased heart rate compared with IN saline. IN epinephrine decreased histamine-induced nasal congestion.

## Background

Anaphylaxis is a serious, life-threatening allergic reaction that is fatal for approximately 186 to 225 people in the United States each year [1]. Rates of severe allergy and anaphylaxis in the United States and other countries have progressively increased over the past 20 years [1–3]. The first choice of therapy for severe allergy and anaphylaxis treatment is epinephrine, typically administered intramuscularly (IM) via an autoinjector [4, 5]. Immediate epinephrine administration is required to reduce allergic or anaphylactic symptoms and hospitalizations and to prevent fatal outcomes [4–7].

Despite the effectiveness of epinephrine delivery via autoinjector, issues or concerns associated with autoinjector use exist [8, 9]. Use of an autoinjector is often avoided or delayed because of user anxiety surrounding stigma or fear of self-harm, especially in children and adolescents [8–10]. In children, there is an increased risk of intraosseous administration and muscle laceration with autoinjector use [8, 11]. Lack of portability is also a concern, and patients prescribed autoinjectors do not always carry them [12, 13]. Understanding patients’ perspectives on carrying frequency, confidence in use, and training experiences may impact the likelihood of a patient having his or her medication on hand in the event of an anaphylactic emergency [12].

The intranasal (IN) route of administration is an alternative option for the treatment of severe allergy and anaphylaxis and offers several potential advantages over the IM route [14]. IN administration allows for a noninvasive and convenient mode of drug delivery via self-administration [14]. The nasal cavity provides an environment for rapid drug absorption because of high vascularization and tissue permeability, which allow for a shorter time to onset of effect and higher bioavailability [15]. Also, avoidance of the gastrointestinal tract and hepatic metabolic breakdown increases drug availability [15]. IN administration has been explored with drug formulation design for the treatment of conditions other than anaphylaxis, including pain (opioids), heroin overdose reversal (opioid antagonists), Alzheimer’s disease, and seizures (benzodiazepines) [16–19].

Though IN delivery has several benefits, alterations in the nasal environment may influence drug absorption and delivery to target areas. Nasal congestion has been reported during anaphylactic episodes, and has the potential to interfere with IN epinephrine in the treatment of anaphylaxis [20, 21]. During an anaphylactic or allergic event, the release of inflammatory mediators, such as histamine, contribute to vasodilation [22, 23]. Increased vasodilation can result in edema and swelling of the nasal mucosa, impeding air flow and increasing nasal secretions [22, 23]. However, alpha-adrenergic receptor agonists like epinephrine have known decongestant activity [24]. During an allergic response, histamine release may counteract the vasoconstrictive effects of epinephrine. Histamine may in turn accentuate, rather than impair, epinephrine absorption after IN epinephrine administration. In one preclinical study, IN epinephrine paired with IN phentolamine, a vasodilator, led to a more pronounced increase in plasma epinephrine concentration as compared with IN epinephrine administration alone [25].

The aim of this study was to investigate the impact of nasal congestion induced by histamine administration on the systemic absorption of epinephrine after IN epinephrine administration. We hypothesized that nasal congestion would not interfere with epinephrine absorption following IN epinephrine administration.

## Methods

### Dogs

Experiments were approved by the Institutional Animal Care and Use Committee of MRIGlobal (Kansas City, MO, USA) before dog procurement from a United States Department of Agriculture (USDA)-certified vendor (Covance Research Products, Denver, PA). Dogs were individually housed indoors in primary enclosures (cage banks, Shor-line) that provided floor space either meeting or exceeding specifications of the USDA Animal Welfare Act and as described in the *Guide for the Care and Use of Laboratory Animals* [26]. Dogs were housed under controlled environmental conditions with a standard 12-h light/dark cycle, provided free access to food and water, exercised thrice weekly (≥90 min), and received daily positive interaction from MRIGlobal staff.

### Anesthesia

After 15 h of fasting, dogs were sedated with IM buprenorphine (0.01 mg/kg). Anesthesia was induced intravenously with 6 mg/kg propofol and maintained with inhaled isoflurane and oxygen at 2 L/min. Dogs were monitored every 5 min for pulse oximetry, blood pressure, electrocardiogram activity, heart rate, respiratory rate, body temperature, mucous membrane color, and capillary refill time. Intravenous fluids (0.9% saline solution) were administered at 5 mL/kg/hour. Additional boluses at 10 mL/kg increments were administered if hypotension (mean arterial blood pressure < 60 mmHg) developed. After procedure completion, isoflurane was stopped, and the dogs breathed oxygen for up to 5 min before swallow reflex onset and extubation.

### Formulation

A solution of 5% histamine (Sciencelab.com, lot #SLH1099) was formulated on study Days 0 and 2, stored at 20–25 °C, and protected from light. Two grams of histamine were added to 40 mL of sterile 0.9% saline (Hospira, lot #89–617-FW) and mixed until completely dissolved.

Epinephrine was purchased from Spectrum Chemical Manufacturing Corp. (St. Louis, MO), stored at 5 ± 3 °C, and protected from light. The vehicle for epinephrine was formulated at MRIGlobal and was based on the injectable product with appropriate modifications suitable for IN administration. In addition to water for injection, sodium metabisulfite (SMBS), and sodium chloride, the formulation included a viscosity modifier, preservative, and buffer. The final formulation had a pH of 5.0 ± 0.5.

### Change in nasal pressure

**Fig. 1:**
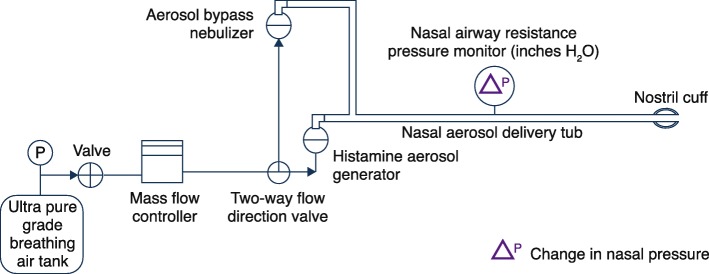
Intranasal Aerosol Delivery and Pressure Measurement System. Schematic of the system used for measurements of changes in nasal congestion restriction

We first confirmed the time course of histamine-induced changes in nasal restriction flow pressure that was previously described by Tiniakov and colleagues [27] (Supplementary Figure 1). Histamine was delivered via an IN aerosol delivery and pressure measurement system based on the previously developed model [27] (Fig. 1). Controlled air supply (Praxair Inc., Danbury, CT) provided pressure to a calibrated flow controller (Sierra Instruments, Inc., Monterey, CA). The flow controller metered the air at a set flow rate to the nebulizer (Philips Respironics SideStream) for histamine aerosol generation. A 3.5-mm endotracheal cannula (Teleflex, Inc., Wayne, PA) with a calibrated digital pressure monitor (475 Mark III, Dwyer Instruments, Inc., New Britain, PA) was inserted to measure real-time changes in nasal pressure. At 10 min after cannulation into the left nostril, all dogs received 5% histamine IN over 5 min. Changes in nasal pressure were measured in inches of water (in. H_2_O) at 0, 5, 10, 15, 20, 91, 100, 105, 110, 115, 122, 124, 128, and 130 min.

**Fig. 2:**
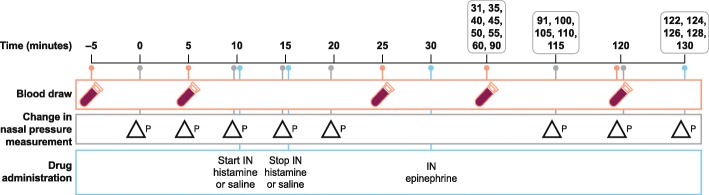
Study Design. Timing of nasal pressure measurements and pharmacokinetic measurements after histamine or saline IN administration, following epinephrine IN administration

The current study was conducted to evaluate the impact of IN epinephrine on epinephrine pharmacokinetics (PK) and heart rate after IN histamine versus saline. The study was conducted over 4 days using the IN aerosol delivery and pressure measurement system described above. At 10 min after cannulation into the left nostril, dogs received either 5% histamine or saline IN over 5 min; 15 min later, 4 mg epinephrine IN was administered into the same nostril. Changes in nasal pressure were measured in inches of water (in. H_2_O) at 0, 5, 10, 15, 91, 100, 105, 110, 115, 120, 122, 124, 128, and 130 min (Fig. 2).

### Pharmacokinetic analysis

Epinephrine plasma concentrations were measured in blood samples taken at − 5, 5, 25, 31, 35, 40, 45, 50, 55, 60, 90, and 120 min (Fig. 2). Plasma samples were vortex-mixed for approximately 1 min, followed by centrifugation and aliquoting (at least three 100 μL aliquots per sample) into microcentrifuge tubes containing SMBS (5 μL) on ice and protected from light. The aliquots (one per sample) were transferred to the Bioanalytical Group for analysis. Remaining bulk plasma and aliquots were stored at − 80 °C.

Control plasma was heat-treated (55 °C for approximately 8 days) and stabilized with SMBS (317 mg/mL; plasma/SMBS = 98:2, v/v). Calibrators, quality control samples, blanks, and test samples were prepared by solid-phase extraction using Biotage Evolute Express WCX 96-well plates and epinephrine-d6. Liquid chromatography with tandem mass spectrometry was performed in positive electrospray ionization mode using multiple reaction monitoring ionization. The calibrator range was set between 1 and 32 ng/mL, with quality control samples of 4, 12, and 24 ng/mL, or 0.4–10 ng/mL, with quality control samples of 1, 3, and 5 ng/mL.

PK data analysis was performed using Phoenix32 WinNonlin software (Version 8.1; Pharsight Corporation, St. Louis, MO, USA). The average concentrations of three predose samples were subtracted from the postdose measurement for each dog. Samples were assigned a value of zero if a negative value was calculated after baseline subtraction. Outliers exceeding two times the standard deviation (SD) from the respective baseline-adjusted concentrations were removed from analysis. PK parameters included maximum concentration (C_max_), time to reach C_max_ (T_max_), and area under the plasma concentration-time curve (1–90 min) (AUC_1–90_). The trapezoid rule was used to calculate the AUC_1–90_, and student’s *t*-tests were used to compare parameters between study groups. Statistical significance was defined as *p* < 0.05. Bioequivalence was assessed using log-transformed C_max_, T_max_, and AUC_1–90_ of individual dogs. Bioequivalence was defined as the 90% confidence interval (CI) of the geometric mean ratio between the histamine and saline groups between 80 and 125%.

### Heart rate

Heart rate data were collected via the DRE Waveline VS (DRE, Inc., Louisville, KY, USA) and recorded at − 5, 0, 5, 10, 15, 20, 25, 30, 31, 35, 40, 45, 50, 55, 60, 90, 91, 100, 105, 110, 115, 120, 122, 124, 126, 128, and 130 min.

### Clinical observations

Clinical observations were reported in the morning and evening on all days except the dosing day. On study Days 0–3, clinical observations were reported throughout the anesthetic episode and into recovery. Normal post-study daily observations were conducted to confirm that normal functions and activity levels were restored in all dogs.

## Results

### Change in nasal pressure

**Fig. 3:**
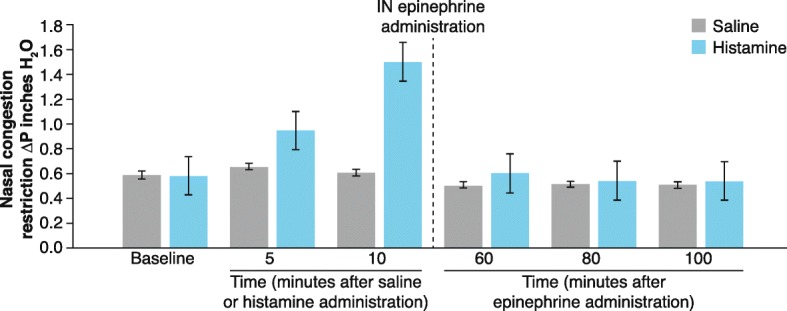
Effect of IN Epinephrine on Histamine-Induced Nasal Congestion. IN histamine led to increased nasal pressure at 10 min after administration, as compared to IN saline. IN epinephrine reduced histamine-induced nasal pressure increase at all time points measured. Data represent mean ± SEM. IN, intranasal, P, pressure

The current study consisted of two treatment groups: histamine/epinephrine IN (*n* = 6) and saline/epinephrine IN (*n* = 6) (Table 1). Male and female dogs were between 10 and 13 months of age and weighed approximately 7–14 kg. At baseline, the average nasal pressure was 0.58 in. H_2_O for both histamine and saline groups. At 5 and 10 min, nasal pressure was increased for the histamine group compared with the saline group (mean ± standard error of the mean [SEM], 5 min: 0.94 ± 0.20 vs 0.65 ± 0.04 in. H_2_O, *p* = 0.19; 10 min: 1.49 ± 0.47 vs 0.61 ± 0.04 in. H_2_O, *p* = 0.09) (Fig. 3). IN epinephrine reduced histamine-induced nasal congestion to nasal restriction pressure levels comparable to those of saline at 60, 80, and 100 min after epinephrine (mean ± SEM, 60 min: 0.60 ± 0.11 vs 0.51 ± 0.03 in. H_2_O, *p* = 0.43; 80 min: 0.54 ± 0.08 vs 0.51 ± 0.03 in. H_2_O, *p* = 0.78; 100 min: 0.54 ± 0.07 vs 0.51 ± 0.02 in. H_2_O, *p* = 0.69) (Fig. 3).

**Table 1:** Drug Administration

Drug administration	n
5% histamine, followed by epinephrine 4 mg IN	6
0.4–0.7 mL saline, followed by epinephrine 4 mg IN	6

*IN* Intranasal

### Pharmacokinetics

**Fig. 4:**
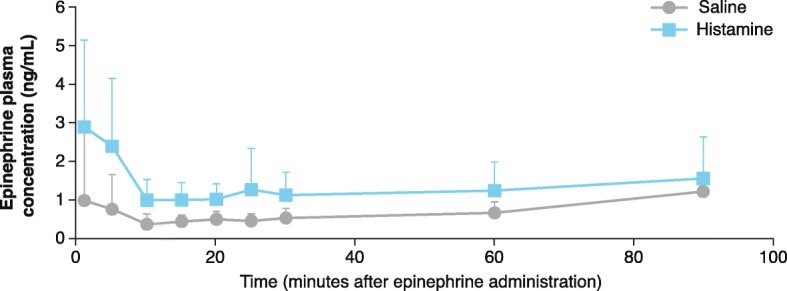
Effect of IN Epinephrine on Epinephrine Plasma Concentrations After IN Histamine or Saline. Group average epinephrine concentration-time profiles are plotted for the histamine and saline groups. Immediately after IN epinephrine, the average epinephrine plasma concentration was greater in the histamine group versus the saline group. Data represent mean ± SD. IN, intranasal

**Fig. 5:**
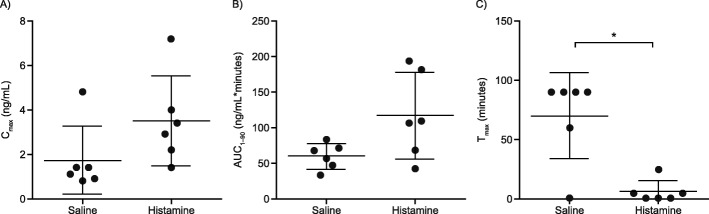
Effect of IN Epinephrine on Epinephrine Pharmacokinetics After IN Histamine or Saline. While there were no statistically significant differences in Cmax and AUC_1–90_ between groups, there was a significantly shorter T_max_ with the histamine versus saline group. The reported C_max_ and T_max_, values were calculated using post-dose baseline-subtracted epinephrine concentrations for each dog, and the AUC_1–90_ was calculated using the trapezoid rule. Plasma concentration vs time data were first analyzed for each individual dog, and then PK parameters were averaged from individual dogs within each group. Data represent mean ± SD. *p < 0.05. AUC_1–90_, area under plasma concentration-time curve from 1 to 90 min; C_max_, maximum concentration; IN, intranasal; PK, pharmacokinetics; T_max_, time to reach maximum concentration

An immediate increase in plasma epinephrine concentration was observed after IN epinephrine administration in the histamine group versus the saline group (Fig. 4). There were no significant differences in C_max_ and AUC_1–90_ between the histamine and saline groups after administering IN epinephrine (mean ± SD, 3.5 ± 2.1 vs 1.7 ± 1.5 ng/mL, *p* = 0.06 and 117 ± 61.0 vs 59 ± 18.0 ng/mL*minutes, *p* = 0.09, respectively) (Fig. 5a, b). After IN epinephrine delivery, there was a significantly lower T_max_ with the histamine versus saline group (mean ± SD, 6 ± 9.0 vs 70 ± 36.0 min; *p* = 0.02) (Fig. 5c).

IN epinephrine did not demonstrate bioequivalence between dogs who received histamine versus those who received saline. The ratio of geometric means (90% CI) were as follows: C_max_, 217 ng/mL (115–408); T_max_, 7.4 min (1.4–3.9); and AUC_1–90_, 180 ng/mL*minutes (109–296).

### Heart rate

**Fig. 6:**
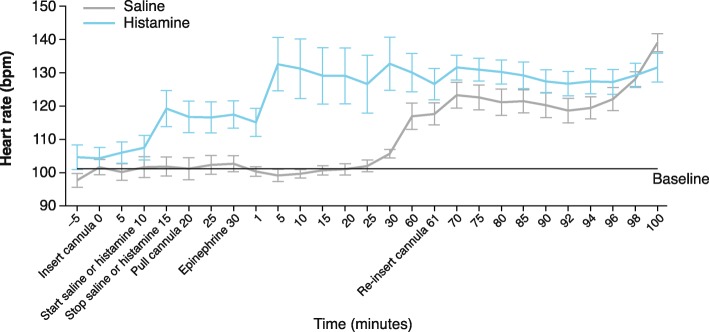
Effect of IN Epinephrine on Heart Rate After IN Histamine or Saline. Mean heart rate immediately increased after IN histamine versus IN saline administration. At 5 min after IN epinephrine delivery, mean heart rate increased in the histamine group, and remained near baseline levels in the saline group. Elevations in heart rate were maintained in the histamine group, and occurred in the saline group at 60 min, and then maintained for the duration of the study. Data represent mean ± SD. bpm, beats per minute, IN, intranasal

The mean ± SD baseline heart rates were 104 ± 18.1 beats per minute (bpm) and 97 ± 10.1 bpm for the histamine and saline groups, respectively. An immediate increase in heart rate was observed following IN histamine as compared with IN saline administration (119 ± 26.6 and 102 ± 14.1 bpm, respectively) (Fig. 6). At 5 min after IN epinephrine delivery, heart rate in the histamine group increased to 132 ± 39.4 bpm, whereas heart rate remained near baseline levels in the saline group (99 ± 9.1 bpm). At 60 min after IN epinephrine, heart rate was maintained at 130 ± 28.3 bpm in the histamine group and increased to 117 ± 19.4 bpm in the saline group. Elevations in heart rate were maintained in both the histamine and saline groups through 100 min after IN epinephrine administration.

### Clinical observations

Clinical observations included, but were not limited to, abnormal fecal observations, decreased food consumption, emesis, excessive salvation, and alterations in activity. Dogs underwent and recovered from all anesthetic episodes without incidence and did not display any signs of pain or distress. There were no adverse clinical observations reported during this study.

## Discussion

In the current study, IN epinephrine led to faster epinephrine absorption and more rapidly increased heart rate in the IN histamine group. IN epinephrine led to faster absorption of epinephrine in the histamine group, as indicated by the significantly decreased T_max_ in the IN histamine group. The effect of more rapidly increased epinephrine absorption following IN epinephrine in the histamine group may be due to the known vasodilatory effects of histamine [22]. It is likely that histamine-induced vasodilation offsets the vasoconstrictive properties of IN epinephrine, resulting in enhanced absorption.

IN epinephrine resulted in increased plasma epinephrine, and the increase in epinephrine absorption was further enhanced when paired with histamine-induced nasal congestion. Dretchen and colleagues have shown in dogs that IN epinephrine produces more rapidly heightened average plasma epinephrine concentration versus IM epinephrine (1 min versus 5 min post-administration) [28]. To our knowledge, the only other preclinical studies of IN epinephrine, also in dogs, showed that IN epinephrine-induced effects on epinephrine concentration were more pronounced when IN epinephrine was administered along with a nasal decongestant [25, 29]. The only clinical study on IN epinephrine was a pilot study in 5 participants, which found that similar epinephrine absorption occurred after IN versus IM epinephrine [30]. Future clinical studies are needed to address the effects of nasal congestion on epinephrine absorption following IN epinephrine administration.

IN epinephrine led to immediate increases in heart rate, in line with rapidly increased plasma epinephrine, in the histamine group. After IN epinephrine administration, heart rate increased at a quicker rate at 5 min in dogs who received histamine versus those who received saline, in which heart rate elevations started 30 min post IN epinephrine. In dogs, IN epinephrine paired with a nasal decongestant led to increased epinephrine-induced effects on cardiovascular parameters, including coronary perfusion pressure, as compared to IN epinephrine alone [25, 29]. The potential vasodilatory effects of histamine paired with the decongestant effects of epinephrine may lead to an accelerated onset of pharmacodynamic effects of IN epinephrine, as indicated by the prompt epinephrine absorption and rapid increase in heart rate following IN epinephrine in the histamine group.

There are several limitations of this study. Human studies will be required to evaluate the absorption of IN epinephrine during nasal congestion, as there are potential differences in PK and pharmacodynamic responses, as well as drug delivery, in dogs versus humans. In addition, there were increases in heart rate almost immediately following IN administration of histamine, followed by a second increase after epinephrine administration. This second peak level was maintained throughout the experiment and may have occurred as a result of the isoflurane anesthetic. Also, the effects of epinephrine on heart rate were not compared with those of saline alone after histamine or saline administration, as both the histamine and saline groups received epinephrine. Lastly, there was not a comparison to the EpiPen autoinjector, which is most commonly used in the treatment of anaphylaxis.

IN epinephrine may offer convenience with its small size, ease of carrying, safety, and noninvasiveness as compared with the autoinjector [15]. A treatment method like IN delivery that is easier and more convenient may lead to increased compliance and prompt epinephrine administration in patients during severe allergy or anaphylaxis [12].

## Conclusions

IN epinephrine decreased the nasal congestion induced by IN histamine administration. IN epinephrine produced more rapid onset of epinephrine absorption and increased heart rate in dogs who received IN histamine. Therefore, nasal congestion does not inhibit IN epinephrine administration, nor does it hinder epinephrine absorption. The IN route is a potential alternative to the IM route for epinephrine administration in the treatment of anaphylaxis. Clinical studies comparing IN epinephrine in the context of nasal congestion should be considered to help validate the results of this study. Future approaches will aim to evaluate IN epinephrine in clinical studies. These future studies will evaluate epinephrine PK and PD effects after IN versus IM administration in humans.

## Supplementary information

**Additional file 1 **: **Figure S1**. Effect of IN Histamine on Nasal Pressure. At baseline, the average nasal pressure was 0.55 in. H_2_O. Five minutes following IN histamine administration, nasal pressure increased to 0.73 in. H_2_O. The average change in nasal pressure was greatest at 60 min after histamine administration (1.11 in. H_2_O), and nasal pressure remained heightened for up to 80 and 100 min after histamine administration (1.10 and 0.96 in. H_2_O, respectively). Data represent mean ± SEM. IN, intranasal; P, pressure.

## Data Availability

The datasets generated during and/or analyzed during the current study are available from the corresponding author on reasonable request.

## Abbreviations

AUC_1–90_: Area under plasma concentration-time curve from 1 to 90 min
CI: Confidence interval
C_max_: Maximum concentration
IM: Intramuscular
IN: Intranasal
PK: Pharmacokinetic
SBMS: Sodium metabisulfite
SD: Standard deviation
T_max_: Time to reach C_max_
